# NCOA4 and ferritinophagy in hematological malignancies: a double-edged regulator of iron metabolism and cell fate

**DOI:** 10.3389/fonc.2025.1717435

**Published:** 2026-01-20

**Authors:** T. Chabane, D. Bouscary, E. Grignano

**Affiliations:** 1 Université de Paris, Institut Cochin, CNRS UMR 8104, INSERM U1016, Paris, France; 2 Assistance Publique-Hôpitaux de Paris, Centre-Université de Paris, Service d’Hématologie clinique, Hôpital Cochin, Paris, France; 3 Division of Hematology, Department of Medicine, McGill University, Montreal, QC, Canada

**Keywords:** acute myeloid leukemia, CHIP, ferritinophagy, ferroptosis, iron, NCOA4, reactive oxygen species, Tet2

## Abstract

Ferritinophagy, a selective autophagic process mediated by NCOA4, plays a central role in cellular iron homeostasis by mobilizing iron from ferritin to sustain mitochondrial metabolism and redox balance. In cancer, ferritinophagy’s effects vary with context: it can support metabolic fitness in some settings while promoting ferroptotic vulnerability in others. In acute myeloid leukemia (AML), evidence suggests that leukemic stem cells rely more heavily on iron-driven mitochondrial metabolism, making ferritinophagy a potential therapeutic target. This review summarizes current knowledge of NCOA4 regulation and ferritinophagy, discusses their relevance in hematologic malignancies, and highlights therapeutic opportunities and unresolved questions in AML.

## Introduction

Ferritinophagy, the selective autophagic degradation of ferritin, is essential for maintaining intracellular iron homeostasis. This process is mediated by the cargo receptor Nuclear Receptor Coactivator 4 (NCOA4). It releases labile iron (Fe²^+^) as needed by the cell. Iron availability centrally regulates hematopoiesis by supporting mitochondrial metabolism, heme synthesis, and iron–sulfur (Fe–S) cluster formation in hematopoietic stem and progenitor cells (HSPCs) and erythroid precursors (1). Genetic disruption of Fe–S assembly or iron trafficking impairs erythropoiesis and HSC function (2). NCOA4-mediated ferritinophagy is a key source of bioavailable Fe²^+^. It enables hemoglobinization and iron recycling in erythroblastic islands (3, 4). Iron metabolism, Fe–S cluster biogenesis, and ferritinophagy form an integrated network that maintains hematopoietic homeostasis and enables metabolic adaptation during stress or lineage commitment. In leukemic transformation, malignant blasts and leukemic stem cells (LSCs) depend more on ferritinophagy to meet increased bioenergetic and anabolic demands. This reliance lowers the threshold for ferroptosis under oxidative stress (5). Such hematopoietic-specific vulnerabilities illustrate why NCOA4 dysregulation affects leukemogenesis more than solid tissues. While ferritinophagy is essential for normal physiology, such as erythropoiesis, its roles in cancer are context-dependent. Depending on metabolic and redox constraints, ferritinophagy may support tumor growth by supplying iron or promote ferroptosis-driven cell death. This dual role emphasizes its importance in oncology. Also, beyond ferritinophagy, NCOA4 acts as a nuclear receptor coactivator and influences DNA replication (6). NCOA4 shows dynamic localization during mitosis, associating with microtubules and midbody structures. This suggests possible roles in microtubule dynamics, chromosome segregation, or cytokinesis (7). NCOA4 also modulates innate immune crosstalk, for example, in anti-infection responses (8).

This review synthesizes findings from these sources and integrates them to focus on the roles of NCOA4 and ferritinophagy in iron metabolism and tumorigenesis, with particular attention to their recently uncovered impact in hematological malignancies.

## Ferritinophagy in iron metabolism

Iron is essential for many biological processes, including DNA synthesis, mitochondrial respiration, heme/Fe–S cluster biosynthesis, and oxygen transport. To avoid iron toxicity, cells store excess iron in ferritin, a 24-subunit complex composed of ferritin heavy chain (FTH1) and ferritin light chain (FTL). Ferritin sequesters iron in a redox-inert form (Fe³^+^), thereby preventing uncontrolled ROS production via Fenton chemistry. However, ferritin-bound iron is metabolically unavailable unless it is mobilized through degradation pathways.

For decades, the mechanism by which cells mobilize stored iron remained unclear. This understanding changed in 2014, when Mancias et al. (9) and Dowdle et al. (10) independently identified NCOA4 as a selective cargo receptor for ferritin, laying the foundation for current research on ferritinophagy.

Ferritinophagy is a selective form of autophagy that degrades ferritin (FTH1/FTL complex) to release ferrous iron (Fe²^+^) into the cytosol. NCOA4 mediates this process by serving as a cargo receptor linking ferritin to the autophagy machinery for lysosomal degradation. NCOA4 lacks a typical LIR domain but uses an alternative motif to interact with ATG8 family proteins (11). This degradation releases the stored iron into the cell’s labile iron pool (LIP), making it available for crucial biological processes. Intracellular iron levels tightly regulate NCOA4 expression and stability. When iron is abundant, the E3 ubiquitin ligase HECT and RLD domain-containing E3 ubiquitin protein ligase 2 (HERC2) ubiquitinates NCOA4. This marks NCOA4 for proteasomal degradation to prevent excessive iron release and potential toxicity. In hematologic malignancies, iron homeostasis is often perturbed. This is especially true in MDS and secondary AML, where ineffective erythropoiesis and transfusional iron overload are common. Under these conditions, altered intracellular iron levels may influence HERC2-dependent regulation of NCOA4 stability. NCOA4 ubiquitination is iron-sensitive. Although direct evidence in primary leukemic cells is limited, this framework plausibly links disease-associated iron overload to altered ferritinophagy flux. Conversely, under iron-deficient conditions, NCOA4 levels rise to promote ferritinophagy and replenish the LIP. Recently, Kuno et al. (12) showed that NCOA4 regulates ferritin fate under iron-replete conditions. Its intrinsically disordered regions and C-terminal domain promote Fe(III)-dependent condensate formation (~120 nm in diameter). These condensates sequester NCOA4 away from ferritin, thereby stabilizing ferritin during the early phases of iron repletion. With prolonged repletion, Tax1 Binding Protein 1 (TAX1BP1) binds NCOA4 and directs ferritin to lysosomes through an ATG7-independent aggrephagy pathway. This prevents excessive iron sequestration and iron deficiency. By controlling the balance between ferritin-bound iron and LIP, ferritinophagy plays a central role in iron homeostasis. This regulation is fundamental. Excess labile iron is highly reactive and can generate toxic reactive oxygen species (ROS) via the Fenton reaction, a key driver of ferroptosis (13). The NCOA4-ferritinophagy axis thus serves as a central regulator of the cell’s sensitivity to ferroptosis. Besides, ferritinophagy is also crucial for erythropoiesis *in vivo*, as it mobilizes iron from ferritin for use in heme synthesis (3).

## Ferritinophagy, ferroptosis and cancer

Ferritinophagy demonstrates a dual role in carcinogenesis. On the one hand, it promotes tumor growth by releasing Fe²^+^ from ferritin, expanding the labile iron pool, and supporting biosynthetic pathways vital for cancer cell growth—including DNA synthesis, mitochondrial respiration, Fe–S cluster biosynthesis, and heme biosynthesis. When antioxidant defenses are intact, this mobilized iron enables proliferation, invasion, and adaptation of iron-addicted tumors. On the other hand, excessive iron flux that overwhelms antioxidant systems increases susceptibility to ferroptosis, thus acting as a mechanism of tumor cell death. This duality means that ferritinophagy can both fuel cancer cell survival and sensitize cells to iron-dependent cell death, depending on the cellular context.

On the other hand, if iron flux overwhelms the antioxidant system—such as when GPX4 or xCT function is compromised—ROS and lipid peroxides accumulate. This leads tumor cells to undergo the lethal process of ferroptosis.

Ferroptosis is an iron-dependent form of regulated cell death driven by lipid peroxidation, as extensively described elsewhere (14). First described in 2012 by Stockwell et al., it is a form of cell death distinct from other regulated death pathways (apoptosis, necrosis, autophagy, pyroptosis) because it directly depends on iron. It is characterized by the accumulation of lipid peroxides in membranes, resulting from the peroxidation of polyunsaturated fatty acids such as arachidonic or adrenic acid, which are incorporated into phospholipids and subsequently oxidized by lipoxygenases. Excess Fe²^+^ amplifies this process through Fenton reactions. These lipid peroxides propagate across membranes in a chain reaction, ultimately causing membrane rupture and cell death (13).

Key studies show that NCOA4 upregulation sensitizes cells to ferroptosis (15), whereas silencing NCOA4 protects them against ferroptotic stimuli. In macrophages, NCOA4-activated ferritinophagy is regulated by the stimulator of the interferon response cGAMP interactor 1 (STING) pathway, thereby accelerating ferroptosis, suggesting a potential role for NCOA4 in immune regulation (16). Mechanistically, ferritinophagy-induced iron release drives ROS accumulation, lipid oxidation, and ferroptotic signaling (17). This dual role—fueling metabolism and sensitizing to ferroptosis—makes ferritinophagy a pivotal regulator of cancer cell fate.

## Ferritin export mechanisms involving NCOA4

Traditionally, ferroportin has been considered the primary iron export pathway and the only known ferrous iron transporter. More recently, however, secreted ferritin has also been proposed as an alternative route of iron export (18), although mammalian ferritin lacks a signal peptide for conventional ER–Golgi secretion. Meyron-Holtz and colleagues demonstrated that ferritin release occurs via non-classical mechanisms, including secretory autophagy and the multivesicular body–exosome pathway, particularly in conditions of impaired endo-lysosomal trafficking (19).

Moreover, beyond its role in ferritin degradation, subsequent studies reported that NCOA4 knockdown or knockout did not decrease serum ferritin levels and even enhanced ferritin secretion under blocked endo-lysosomal or lysosomal-damaged conditions, suggesting that NCOA4 does not participate in secretory autophagy of ferritin (19, 20). However, more recent findings indicate that NCOA4 can facilitate ferritin transfer to CD63, an extracellular vesicle–associated protein, for secretion under iron-loaded conditions. Notably, CD63 expression is regulated by the iron regulatory proteins (IRPs) and iron-responsive elements (IREs) (IRE–IRP) system, activated during iron excess (21). Recent work demonstrates that NCOA4 associates with CD63-positive multivesicular bodies and facilitates ferritin loading into secreted vesicles, providing a mechanism for iron export independent of ferroportin-mediated transport. This process appears particularly relevant under conditions of iron excess or lysosomal stress and has been proposed as an adaptive pathway to limit intracellular iron toxicity (22). Supporting this, another study showed that chloroquine, a lysosomal acidification inhibitor, promotes the release of autophagy receptors, including NCOA4, through single-membrane endosomes and double-membrane compartments (23).

Together, these emerging data point to NCOA4’s involvement in non-canonical ferritin trafficking and export. NCOA4 can mediate ferritin incorporation into multivesicular bodies and exosomes, thereby enabling its extracellular release. This pathway not only regulates intracellular iron levels but also contributes to intercellular iron exchange within the tumor microenvironment. Cancer cells may exploit NCOA4-mediated ferritin export to modulate stromal or immune cell iron availability, influencing angiogenesis, immune evasion, and metastatic potential.

## NCOA4 and ferritinophagy in solid tumors

The role of ferritinophagy varies across cancers, functioning as either pro-tumorigenic or tumor-suppressive. In many solid tumors, NCOA4 dependency supports iron availability and mitochondrial function. In pancreatic ductal adenocarcinoma (PDAC), ferritinophagy is upregulated to sustain tumor growth, and NCOA4 loss delays tumor progression, partly through reduced Fe–S cluster biogenesis. A high ferritinophagy signature also predicts poor prognosis in PDAC (24). Conversely, in some contexts, NCOA4 activation enhances ferroptosis, conferring tumor-suppressive effects. In glioblastoma, ferritinophagy induction triggers ferroptosis (25, 26), while in breast cancer, GPX4 depletion combined with NCOA4 activity suppresses tumor growth (27). This sensitivity is not universal, as colon cancer cells appear less dependent on ferritinophagy, likely due to compensatory iron uptake pathways (28). NCOA4 function can also diverge within tumor types: different isoforms exert opposite effects in ovarian cancer (29), and NCOA4α behaves as a tumor suppressor in prostate cancer (30). Beyond metabolic roles, NCOA4 influences tumor–immune interactions; reduced expression in renal clear cell carcinoma correlates with poor immune infiltration, and activated CD8^+^ T cells enhance lipid peroxidation in tumor cells (31).

Overall, dependency on ferritinophagy varies widely across solid tumors. While iron-addicted malignancies such as PDAC show a clear reliance on NCOA4-mediated iron mobilization, other cancers, including colorectal cancer, appear largely independent, likely due to compensatory mechanisms for iron uptake or storage. Isoform-specific functions of NCOA4 further add complexity, as opposing effects have been reported within the same tumor types, underscoring ferritinophagy as a context-dependent rather than universal metabolic requirement.

In contrast, hematologic malignancies develop within an iron-rich yet tightly regulated environment characterized by ineffective erythropoiesis and transfusion iron overload. Combined with the strong dependence of leukemic stem cells on mitochondrial oxidative phosphorylation and Fe–S cluster biogenesis, this context places ferritinophagy at the center of intracellular iron supply in myeloid neoplasms.

## NCOA4 and ferritinophagy in hematologic malignancies

In the tumor context, altered iron metabolism is now recognized as a key feature of leukemic cells. This imbalance promotes the acquisition of aggressive phenotypes, characterized by uncontrolled proliferation, resistance to apoptotic signals, and the establishment of a deleterious tumor microenvironment.

Specifically, in patients with hematologic disorders, primary iron overload results from ineffective erythropoiesis. This is often compounded by secondary iron overload due to repeated blood transfusions administered to manage anemia. Excessive iron accumulation leads to the generation of reactive oxygen species (ROS), which drive numerous cellular and systemic alterations. ROS can induce DNA damage, including double-strand breaks, thereby contributing to genomic instability and promoting leukemic transformation and progression.

Therefore, we and others have speculated that iron addiction can be leveraged through ferritinophagy to trigger iron-mediated cell death in hematological malignancies, particularly in AML (32). In AML, the balance between iron release and ROS buffering is especially precarious. Iron-dependent mitochondrial remodeling, enhanced oxidative phosphorylation, and high Fe–S cluster demand create a metabolic milieu in which NCOA4-driven ferritinophagy serves as both a survival mechanism and a source of ferroptotic vulnerability. Dihydroartemisinin, an antimalarial compound with documented antitumor activity, relies on ferritinophagy to increase intracellular iron availability and trigger ferroptosis in AML cell lines (33). LSCs are a major cause of therapy resistance and relapse because they can enter a dormant, non-dividing state that makes them refractory to conventional chemotherapy. LSCs exhibit enhanced dependency on NCOA4-mediated ferritinophagy. A detailed study on AML patient-derived xenograft (PDX) models found that the quiescent, LSCs-enriched population (often CD34^+^CD38^-^) has a distinct metabolic profile (34). Specifically, quiescent LSCs show a unique dependence on NCOA4-mediated ferritinophagy. Targeting this pathway showed significant therapeutic potential as genetic or chemical inactivation of NCOA4 impaired LSCs viability and self-renewal *in vivo*, while sparing normal CD34^+^ hematopoietic cells. *In vivo*, treatment with an NCOA4-FTH1 interaction inhibitor reduced the tumor burden and impaired LSCs viability in AML PDX models (34).

A key recent finding is the identification of NCOA4-mediated ferritinophagy as a selective dependency in Tet methylcytosine dioxygenase 2 (Tet2)-deficient hematopoiesis. Loss-of-function mutations in Tet2 are standard drivers of clonal hematopoiesis (CHIP) and myeloid malignancies like myelodysplastic syndromes (MDS) and acute myeloid leukemia (AML). Using an innovative *in vivo* barcoded CRISPR-Cas9 screen, researchers identified NCOA4 as a gene selectively required for the clonal expansion of Tet2-knockout (KO) HSPCs. The study revealed that Tet2-deficient HSPCs exhibit increased mitochondrial mass, cristae density, and ATP production compared with wild-type counterparts. This enhanced mitochondrial activity increases the demand for labile iron, which is required for mitochondrial respiration and Fe–S cluster biogenesis in the electron transport chain. Tet2-KO cells meet this demand by upregulating NCOA4-dependent ferritinophagy. These metabolic changes increase reliance on Fe–S cluster biogenesis and therefore on ferritinophagy as a source of bioavailable Fe²^+^. This reliance creates a synthetic lethal vulnerability that can be exploited by genetic deletion of NCOA4 or by pharmacological sequestration of lysosomal iron, which phenocopies NCOA4 disruption and impairs the competitiveness of Tet2-mutant HSCs (35). In addition, Tet2 loss perturbs α-ketoglutarate–dependent dioxygenases, enzymes that use Fe^3+^ as a cofactor, leading to compensatory shifts in oxidative metabolism that further amplify iron dependence (36). These changes are likely to intersect with canonical iron-sensing pathways such as the IRP/IRE system. While IRP/IRE signaling has not been systematically characterized in Tet2-mutant HSPCs, increased mitochondrial iron demand and redox stress would be expected to favor IRP activation, reduced ferritin translation, and enhanced reliance on ferritinophagy to mobilize intracellular iron.

**Figure 1:**
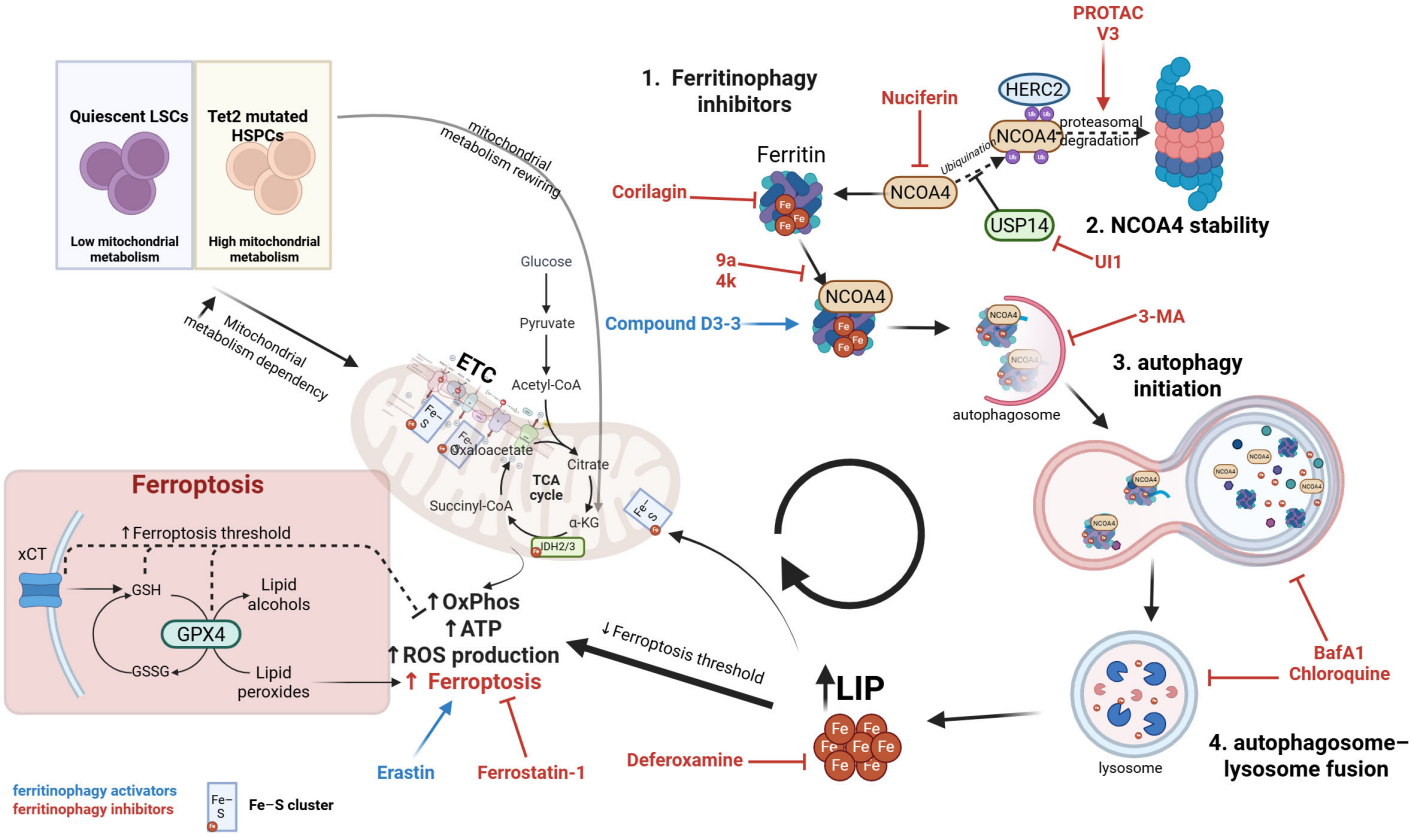
Metabolic and ferritinophagy dependencies in Tet2-mutated HSPCs and leukemic stem cells. Tet2-mutated HSPCs exhibit markedly increased mitochondrial metabolism compared with quiescent leukemic stem cells (LSCs), resulting in elevated oxidative phosphorylation (OxPhos), ATP production, and ROS generation. This heightened bioenergetic state increases dependency on NCOA4-mediated ferritinophagy, which supplies Fe²^+^ to the labile iron pool (LIP) to sustain Fe–S cluster formation and TCA cycle activity. Increased LIP simultaneously lowers the ferroptosis threshold, especially when antioxidant defenses such as the xCT/GPX4 axis are impaired. Pharmacologic modulators act on distinct regulatory nodes of ferritinophagy: (1) Inhibitors of the NCOA4–ferritin interaction (Corilagin, Nuciferine, 9a, 4k) block selective ferritin turnover. (2) Modulators of NCOA4 stability alter iron-responsive ubiquitination, either by promoting proteasomal degradation (PROTAC V3) or inhibiting deubiquitination (UI1 via USP14). (3) Autophagy initiation blockade (3-MA) prevents recruitment of NCOA4–ferritin complexes into autophagosomes. (4) Inhibitors of autophagosome–lysosome fusion (BafA1, Chloroquine) suppress lysosomal ferritin degradation and limit iron release. Conversely, Compound D3-3 (blue) enhances NCOA4-dependent ferritinophagy, increasing LIP. Additional regulators of ferroptosis shown include Erastin (ferroptosis inducer), Ferrostatin-1 (ferroptosis inhibitor), and the iron chelator Deferoxamine, which decreases LIP and mitigates ferroptotic stress. Together, the pathways depicted illustrate how iron mobilization, mitochondrial rewiring, antioxidant defenses, and pharmacologic interventions converge to define a metabolic and iron-dependent vulnerability in Tet2-mutated HSPCs. For clarity, only key regulatory nodes and representative compounds are depicted. All pharmacologic agents shown are currently at a preclinical stage and are included for mechanistic illustration rather than therapeutic validation. *BafA1, Bafilomycin A1; ETC, electron transport chain; Fe–S, iron–sulfur; GPX4, glutathione peroxidase 4; HSPCs, hematopoietic stem/progenitor cells; LIP, labile iron pool; LSCs, leukemic stem cells; OxPhos, oxidative phosphorylation; PROTAC, proteolysis-targeting chimera; ROS, reactive oxygen species; TCA, tricarboxylic acid cycle; USP14, ubiquitin-specific protease 14; xCT, cystine/glutamate antiporter.*.

This dependency suggests that ferritinophagy, as a crucial iron supplier for mitochondrial metabolism, is a critical mechanism supporting the clonal advantage in Tet2-mutated CHIP and LSCs (see **Figure 1**).

## Therapeutic targeting of ferritinophagy

Lowering intracellular ferrous iron levels is a critical strategy for counteracting ferroptosis. However, available chemical approaches to directly target intracellular Fe²^+^ remain limited. At present, iron reduction is mainly achieved with iron chelators such as deferoxamine and deferiprone, which primarily lower total body iron through systemic chelation (13, 37). Yet, because of their mechanism of action, these agents show poor cell membrane permeability and preferentially bind Fe³^+^, making them less effective at directly depleting intracellular ferrous iron.

Given these limitations, the central role of the NCOA4–FTH1 interaction in controlling iron availability and ferroptosis sensitivity makes it an attractive new therapeutic target **(see****Figure 1**). The small molecule 9a is the first-in-class inhibitor that binds directly to NCOA4—specifically its C-terminal domain (aa 383–522) —and disrupts NCOA4 recognition of FTH1, thereby preventing ferritin recruitment to autophagosomes and selectively blocking ferritinophagy while sparing non-selective autophagy. Functionally, 9a reduces the cytosolic labile Fe²^+^ pool without inducing toxic lysosomal iron accumulation, suppressing ferroptosis (38). Consistent with this mechanism, 9a dissociates the NCOA4–FTH1 complex in HEK-293T and HT-1080 cells and lowers labile iron pool (LIP) in HT-22 neurons, and it shows *in vivo* efficacy in a rat ischemia–reperfusion model by limiting lipid peroxidation (39). In acute myeloid leukemia, 9a further exhibits preferential cytotoxicity toward quiescent leukemic stem cells, with comparatively milder effects on proliferating blasts and minimal impact on normal hematopoietic cells (34).

Recent data show that HDAC inhibitors (entinostat, vorinostat) sensitize AML cells to ferroptosis by increasing the labile iron pool through upregulation of iron-metabolism genes, including NCOA4 and HMOX1/2. CRISPR deletion of these genes protects AML cells from HDAC-induced ferroptosis, highlighting an additional mechanism by which ferritinophagy contributes to iron-dependent cell death in AML (40).

Artesunate (ART), an artemisinin derivative, was recently shown to inhibit oxidative phosphorylation (OxPhos) metabolism in AML and LSC-enriched models, enhancing the activity of conventional therapies and overcoming venetoclax resistance while sparing normal HSCs. These findings reinforce the metabolic vulnerability of LSCs and complement earlier evidence linking artemisinin derivatives to iron-dependent oxidative stress (41).

Alternatively, in a rat model of ischemic stroke, pharmacologic inhibition of ubiquitin-specific protease 14 (USP14) with the small-molecule IU1 increased NCOA4 ubiquitination, decreased NCOA4 levels, and consequently alleviated ferroptosis, thereby ameliorating neuronal injury (42).

Rather than simply inhibiting NCOA4 function, degrading the protein itself may be a more effective strategy to block ferritinophagy-driven increases in labile iron completely. Proteolysis-targeting chimera (PROTAC) technology offers a novel therapeutic approach by leveraging the ubiquitin–proteasome system to selectively eliminate disease-associated proteins instead of merely inhibiting them (43, 44). V3 is a VHL-recruiting PROTAC that links an NCOA4-binding ligand to a VHL ligand, bringing NCOA4 to the VHL E3 ligase complex, promoting its ubiquitylation and proteasomal degradation, thereby abolishing NCOA4-mediated ferritinophagy, reducing intracellular Fe²^+^, and suppressing ferroptosis. The mechanism is supported by reversal with proteasome inhibitors, and *in vivo* efficacy has been shown in a mouse model of acute liver injury, with tissue protection accompanied by lowered intracellular Fe²^+^ (45). This targeted degradation strategy offers a promising means to directly regulate iron metabolism by selectively removing NCOA4, though it may raise concerns about off-target effects.

Beyond direct NCOA4 blockade or degradation, several small molecules can attenuate ferritinophagy through distinct mechanisms. Corilagin, an ellagitannin derivative, appears to bind FTH1 and competitively displace NCOA4 from the NCOA4–FTH1 interface, thereby stabilizing ferritin, reducing LIP, and attenuating ferroptosis. Efficacy was demonstrated *in vivo* in a mouse intestinal ischemia–reperfusion model, with tissue protection accompanied by reduced ferroptosis signatures (46). Nuciferine similarly inhibits NCOA4-dependent ferritinophagy, stabilizes ferritin, and protects from ferroptosis in cochlear hair-cell contexts (e.g., under RSL3 exposure), consistent with a decrease in LIP (47). The autophagy inhibitor 3-methyladenine (3-MA) blocks class III PI3K–dependent autophagosome formation, thereby indirectly reducing ferritinophagy and limiting ferritin-derived iron release; this effect has been reported across cell-based models of macro-autophagy (48). Other non-specific autophagy inhibitors, such as chloroquine or Bafilomycin A1 (BafA1), by blocking autophagolysosome fusion or lysosome acidification (for the latter), may mitigate ferritinophagy (49, 50).

Finally, a recent tool compound, termed “Ferritinophagy inhibitor 4k,” is reported to bind NCOA4 and disrupt the NCOA4–FTH1 interface, resulting in lower intracellular Fe²^+^ and protection from ferroptosis. A 2025 report describes a “4k” ferritinophagy inhibitor consistent with this mode of action (51).

Conversely, in an effort to leverage ferritinophagy dependency and iron addiction in tumor cells, the compound D3-3, a derivative of sinomenine, was recently identified as a direct inducer of ferritinophagy: it enhances the FTH1–NCOA4 interaction, promotes Fe²^+^ release and lipid ROS production, leading to autophagy-dependent ferroptosis and inhibition of tumor growth in a colorectal cancer model (52). As shown in **Figure 1**, ferritinophagy modulators target distinct regulatory nodes—including NCOA4 stability, ferritin binding, autophagy initiation, and lysosomal fusion—highlighting multiple intervention points in Tet2-mutant iron dependency.

Despite the growing interest in modulating ferritinophagy, several challenges remain. First, specificity is limited: most inhibitors disrupt autophagy more broadly or affect systemic iron metabolism. Second, the risk of exacerbating systemic iron deficiency or promoting toxic iron sequestration must be considered. Third, targeting NCOA4 may impact erythropoiesis and immune function, necessitating careful dose design and biomarker development. Finally, the pharmacokinetic properties of ferritinophagy modulators remain poorly characterized *in vivo*.

Overall, issues related to specificity, pharmacokinetics, tissue distribution, and potential effects on normal iron homeostasis remain largely unexplored, underscoring the need for cautious interpretation and further validation.

All compounds described in this manuscript are preclinical and included for mechanistic illustration.

They are listed in **Tables 1a**, **1b**.

**Table 1a:** Ferritinophagy inhibitors and their mechanisms of action.

Compound	Mechanism	Model	Reference
Corilagin	Competitively disrupts the FTH1–NCOA4 interaction	Intestinal ischemia/reperfusion injury	Wang et al., 2023
Nuciferine	Downregulates Ncoa4, Fth1, Ftl mRNA	Cisplatin-induced ototoxicity	Gao et al., 2024
Compound 9a	Directly blocks NCOA4 (aa 383–522) binding to FTH1	Ischemic stroke; AML	Fang et al., 2021; Larrue et al., 2024
PROTAC-V3	PROTAC-induced NCOA4 degradation	Acute liver injury	Ji et al., 2024
IU1	Inhibits USP14 → increases NCOA4 ubiquitination	Ischemic stroke	Li et al., 2021
3-Methyladenine (3-MA)	Blocks autophagosome formation	Ischemic stroke	Guo et al., 2024
Chloroquine	Inhibits lysosomal acidification → ferritin degradation	Ventilation-induced pulmonary fibrosis	Huang et al., 2025
Bafilomycin A1	Inhibits autophagosome–lysosome fusion	—	Zhang et al., 2024

**Table 1b:** Ferritinophagy activators/inducers.

Compound	Mechanism	Model	Reference
D3-3	Enhances NCOA4–FTH1 interaction	Colorectal cancer	Zhu et al., 2024
Rapamycin	Increases autophagic flux (indirect ferritinophagy induction)	Acute lymphoblastic leukemia	Gong et al., 2020
Artesunate	Increases LIP and promotes iron-dependent oxidative stress; reported to enhance ferritin turnover and sensitize AML cells to ferroptosis	AML; LSC-enriched models	Grignano et al, 2023 Balansundaram et al, 2025

## Conclusion

Ferritinophagy has emerged as a central regulator of iron metabolism with profound implications in cancer biology. By mobilizing iron from ferritin into the labile iron pool, this process sustains biosynthetic pathways, mitochondrial function, and cellular proliferation while simultaneously lowering the threshold for ferroptosis. This duality—supporting tumor growth on one hand and sensitizing cells to iron-dependent cell death on the other—underscores the ambivalent nature of ferritinophagy and its potential as a therapeutic target.

In hematologic malignancies, and particularly in AML, ferritinophagy takes center stage. LSCs, which underlie relapses and resistance, display a marked dependency on NCOA4 to maintain iron homeostasis and metabolic fitness. Genetic or pharmacological disruption of NCOA4 selectively impairs LSCs viability while sparing normal hematopoietic progenitors, establishing a therapeutic window of high interest. Likewise, in Tet2-mutated clonal hematopoiesis, NCOA4-mediated ferritinophagy emerges as a critical driver of clonal advantage, linking iron metabolism to mitochondrial remodeling and disease progression.

Beyond intrinsic leukemic cell biology, ferritinophagy also shapes the microenvironment. NCOA4 has also been implicated in ferritin secretion through non-canonical vesicular pathways. This mechanism enables iron exchange between leukemic cells, stromal components, and immune cells within the tumor microenvironment. Hence, ferritinophagy can fuel angiogenesis, modulate immune responses, and reinforce the leukemic niche. Targeting this axis could therefore not only weaken leukemic cells directly but also disrupt the supportive microenvironment that sustains their growth.

Therapeutically, new strategies are emerging. Small molecules such as the NCOA4–FTH1 interaction inhibitor 9a, PROTAC-based degraders, and ferritinophagy inducers illustrate the feasibility of testing pharmacological modulation of this pathway. In AML preclinical models, inhibiting ferritinophagy reduced tumor burden and selectively impaired LSCSs, providing proof of concept for its clinical translation.

Taken together, these findings position NCOA4-driven ferritinophagy as both a vulnerability and an opportunity in the treatment of myeloid malignancies. By targeting ferritinophagy, we may simultaneously suppress the metabolic resilience of leukemic cells, induce ferroptosis under pro-oxidant conditions, and dismantle the iron-dependent crosstalk within the tumor microenvironment. This multifaceted potential highlights ferritinophagy not merely as a metabolic adaptation but as a promising therapeutic axis in the treatment of refractory myeloid diseases.

## Unresolved questions and future directions

Despite growing interest in ferritinophagy and iron metabolism in hematologic malignancies, several key questions remain unresolved. First, the upstream regulatory mechanisms governing NCOA4 expression, stability, and subcellular dynamics in primary AML blasts and LSCs remain incompletely defined. Whether specific oncogenic drivers (e.g., FLT3-ITD, NPM1c, IDH mutations) modulate ferritinophagy directly is unknown. Second, the context-dependent duality of ferritinophagy—supporting metabolic fitness under basal conditions while predisposing cells to ferroptotic stress—remains poorly understood, particularly in the heterogeneous metabolic states that characterize AML subpopulations.

Third, it should be noted that much of the current understanding of ferritinophagy derives from cell line–based systems or non-hematopoietic models, which may not fully recapitulate iron dynamics, niche interactions, or metabolic constraints present in primary human leukemia. This limitation underscores the need for *in vivo* and patient-derived studies to define the actual therapeutic relevance of targeting ferritinophagy.

Finally, the precise contribution of ferritinophagy to LSCs maintenance and its interaction with Tet2 loss, mitochondrial rewiring, and Fe–S cluster biogenesis requires further mechanistic dissection *in vivo*. Fourth, although several pharmacologic modulators of ferritinophagy have been identified, their specificity, bioavailability, safety, and therapeutic window in hematologic malignancies remain largely untested. Finally, integrating ferritinophagy-targeted approaches with existing treatments—such as venetoclax-based regimens, epigenetic therapies, or ferroptosis inducers—raises important questions about optimal combinations, biomarkers of response, and mechanisms of resistance.

Addressing these open questions will be essential to determine whether ferritinophagy represents a tractable and clinically actionable vulnerability in AML and related disorders.

## Glossary

3-MA: 3-Methyladenine
α-KG: Alpha-ketoglutarate
ALL: Acute lymphoblastic leukemia
AML: Acute myeloid leukemia
ART: Artesunate
ATP: Adenosine triphosphate
BafA1: Bafilomycin A1
CHIP: Clonal hematopoiesis of indeterminate potential
CQ: Chloroquine
CRISPR-Cas9: Clustered regularly interspaced short palindromic repeats–CRISPR-associated protein 9
D3-3: Enhancer of NCOA4–FTH1 interaction
DFO: Deferoxamine
DHA: Dihydroartemisinin
DNA: Deoxyribonucleic acid
EVs: Extracellular vesicles
ETC: Electron transport chain
Fe–S: Iron–sulfur (cluster)
Fer-1: Ferrostatin-1
FLT3-ITD: Fms-like tyrosine kinase 3 –
FPN: Ferroportin
FTH1/FTL: Ferritin heavy chain 1/Ferritin light chain
GPX4: Glutathione peroxidase 4
GSH: Glutathione
HDAC: Histone deacetylase
HERC2: HECT and RLD domain-containing E3 ubiquitin protein ligase 2
HMOX1/2: Heme oxygenase 1/2
HSCs: Hematopoietic stem cells
HSPCs: Hematopoietic stem/progenitor cells
IDH: Isocitrate dehydrogenase
IC50: Half maximal inhibitory concentration
IU1: USP14 inhibitor
LIP: Labile iron pool
LSCs: Leukemic stem cells
mRNA: Messenger RNA
MDS: Myelodysplastic syndromes
MVB: Multivesicular body
NCOA4: Nuclear receptor coactivator 4
NPM1c: Nucleophosmin 1c
OxPhos: Oxidative phosphorylation
PROTAC: Proteolysis-targeting chimera
PROTAC-V3: NCOA4-degrading PROTAC molecule
RNA: Ribonucleic acid
ROS: Reactive oxygen species
SLC7A11 (xCT): Cystine/glutamate antiporter
TCA: Tricarboxylic acid cycle
TET2: Ten-eleven translocation methylcytosine dioxygenase 2
TFR1: Transferrin receptor 1
USP14: Ubiquitin-specific protease 14.
